# Outcomes of acute type A aortic dissection repair: Daytime versus nighttime

**DOI:** 10.1016/j.xjon.2021.04.017

**Published:** 2021-05-05

**Authors:** Amer Harky, Sabrina Mason, Ahmed Othman, Matthew Shaw, Omar Nawaytou, Deborah Harrington, Manoj Kuduvalli, Mark Field

**Affiliations:** a Department of Cardiac Surgery, Liverpool Heart and Chest Hospital, Liverpool, United Kingdom; b School of Medicine, Faculty of Health and Life Sciences, University of Liverpool, Liverpool, United Kingdom; c Liverpool Centre for Cardiovascular Science, University of Liverpool and Liverpool Heart and Chest Hospital, Liverpool, United Kingdom

**Keywords:** aorta, dissection, timing of surgery, outcomes, ATAAD, acute type A aortic dissection, CVA, cerebrovascular accident, ICU, intensive care unit, IRAD, International Registry of Acute Aortic Dissections, SAR, specialized aortic rota, TIA, transient ischemic attack

## Abstract

**Objective:**

We sought to report our experience of repairing acute type A aortic dissection (ATAAD) over 21 years during in-hours versus out-of-hours before and after the establishment of specialized aortic service and rota.

**Methods:**

A retrospective analysis of all patients who had ATAAD repair between November 1998 and December 2019 in our center. In-hours were defined as 08:00 to 19:59 hours and out of hours were defined as 20:00 to 07:59 hours.

**Results:**

A total of 286 patients underwent repair of ATAAD. Eighty operations took place during the prerota period (43 operations in hours, 37 out of hours) and 206 operations during the specialized rota period (110 in hours, 96 out of hours). There was no difference in 30-day mortality between the in-hours and out-of-hours groups in either the prerota (23.3% vs 32.4%; *P* = .36) or specialized rota periods (11.6% vs 11.5%; *P* = .94). Mean number of cases per year increased by 83% between the prerota and specialized rota periods. Thirty-day mortality reduced in both the in-hours (23.3% vs 11.6%) and out-of-hours (32.4% vs 11.5%) groups since introduction of the specialized aortic rota.

**Conclusions:**

Outcomes in repair of ATAAD during in-hours and out-of-hours periods are similar when operated on in a specialized unit with a dedicated aortic team. This emphasizes the current global trend of service centralization without particular attention to time of day to operate on such critical cohort patients.

## Graphical abstract

Patients included in this study with their respective outcomes.

**Figure:**
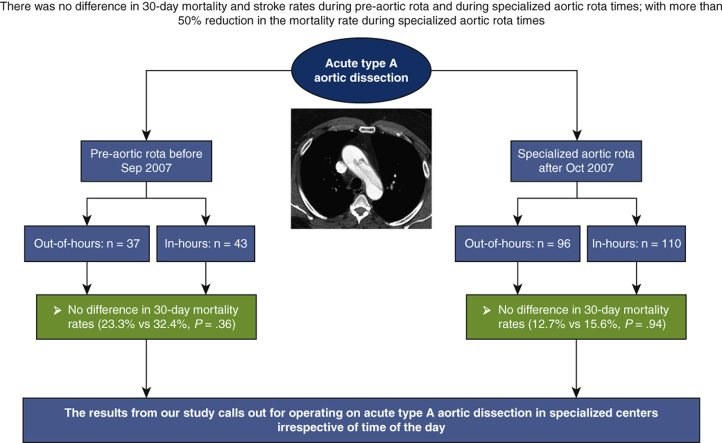

**Figure:**
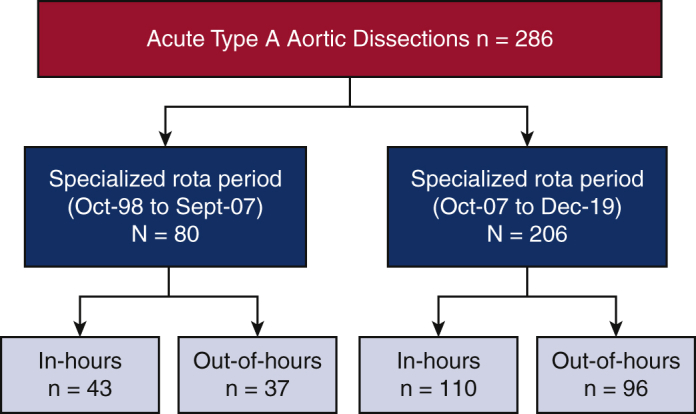
Case distribution between prerota times and specialized aortic rota.

**Central Message**

Surgery for acute type A aortic dissection should not be deferred, irrespective of time of day, provided that experienced, subspecialized aortic teams exist within the hospital.

**Perspective**

It is established that centers with high volume elective aortic practice and subspecialized aortic teams should have no restrictions on timing of operations for emergency acute aortic dissection. Our study compares outcomes of such patients during in-hours and out-of-hours during prespecialized rota and after establishment of specialized rota.

See Commentary on page 21.

Acute type A aortic dissection (ATAAD) is a life-threatening emergency and surgical repair is vital to prevent mortality during both the short term and longer follow-up periods. Guidelines from the European Society of Cardiology,^1^ the American College of Cardiology Foundation, and the American Heart Association^2^ emphasize the importance of immediate surgical intervention to minimize the 50% mortality rate of ATAAD within the first 48 hours if operation is not undertaken. It is by now well established that operating on such patients in high-volume centers in the presence of expertise in aortic surgery and a dedicated team results in, by far, better clinical outcomes.^3^ As such, operating on such patients does not have a merit for timing of surgery, day or night, but rather the presence of such teams. There have been several studies trying to understand the mortality while operating during in hours versus out hours in patients with ATAAD^4^^,^^5^ and many such studies reported debatable outcomes with some of them reporting no significant difference in mortality outcomes, as in the International Registry of Acute Aortic Dissections (IRAD) database.^6^ Therefore, addressing such an important question remains controversial and the purpose of our study was to compare the outcomes of operating on ATTAD during in hours versus out hours during preaortic rota and postaortic rota over the span of 21 years.

## Aims and Objectives

A specialized aortic service and on-call rota was established at the end of 2007 at Liverpool Heart and Chest Hospital, with the aim to reduce rates of mortality and morbidities associated with emergency surgery for ATAAD. The purpose of this study was to investigate the effect of in-hours and out-of-hours surgical intervention for ATAAD on clinical outcomes and survival.

The primary objective was to assess the effect of operative timing on ATAAD repair. We report on operative and postoperative outcomes following in-hours and out-of-hours surgery. We consider in-hours and out-of-hours cases during both the prerota and specialized rota periods.

## Materials and Methods

### Study Population

We reviewed all patients who underwent surgical intervention for ATAAD between October 1998 and December 2019 at Liverpool Heart and Chest Hospital. A specialized aortic service and on-call rota was established in September 2007. Before 2007, all 10 cardiac surgeons participated in a 1:10 on-call rota, doing a few elective aortic operations every year and a few emergency dissections. Acknowledging there is a volume–outcome relationship in complex surgery, the department agreed to center the experience on 3 surgeons at that time. These 3 surgeons did all elective aortic surgery and all ATAAD cases on a 1:3 on-call rota. Over the years and with increasing volumes, this group expanded to 4 aortic surgeons with on-call rota of 1:4 and later to 5 aortic surgeons with 1:5 on-call rota. As such, the specialized aortic service included a significant internal rearrangement, including a dedicated aortic surgeon; recruitment of a devoted aortic fellow; dedicated aortic nursing and theatre staff; and more perfusionists, anaesthetists, and intensivists. Additionally, regional reconfiguration included dedicating our unit as the service center for all major aortic cases in the northwest of England.^7^ Experienced aortic surgeons in our unit are defined as aortic surgeons who perform a minimum of 5 aortic dissections per year and a minimum of 50 cases of thoracic aortic surgeries per year. Additionally, surgeons are required to have 50% of their caseload as elective thoracic aortic surgeries; therefore, there is good exposure and familiarity when operating on emergency cases. Our patients were divided into 2 time periods: a prerota period and a specialized aortic rota (SAR) period, both were then categorized as having had in-hours or out-of hours treatment. In-hours cases were defined as having had operation start times between 08:00 and 19:59 hours, out-of-hours treatment from 20:00 and 07:59 hours; the timing of the shifts is coming from the standard working pattern of the clinical staff at our unit.

### Study Design

Data were collected retrospectively from the hospital's electronic patient record system. All data fields were collected as part of routine clinical practice. This study was approved by the research committee on February 16th, 2021, and the need for patient consent has been waived considering the retrospective nature of the study and no patient-identifiable information is included. Clinical measures record included patient demographic characteristics, intraoperative data, and postoperative outcomes. Patient demographic characteristics included a past medical history of diabetes, hypertension, ischemic heart disease or previous cardiac surgery, and time from presentation to surgery. Intraoperative data was collected on time of operation, extent of aortic repair, time on cardiopulmonary bypass, aortic crossclamp time, and duration of circulatory arrest. The postoperative outcomes evaluated in this study were cerebrovascular accident (CVA), transient ischemic attack (TIA), reoperation for bleeding, new onset renal failure, 30-day mortality, length of intensive care unit (ICU) stay, and total length of hospitalization.

### Definitions

Outcomes were reported based on the definitions set in place by The Society of Thoracic Surgeons Adult Cardiac Surgery Database.^8^ CVA is defined as an acute episode of focal or global neurological dysfunction caused by brain, spinal cord, or retinal vascular injury as a result of hemorrhage or infarction, where the neurological dysfunction lasts >24 hours. TIA is defined as a transient episode of focal neurological dysfunction caused by brain, spinal cord, or retinal ischemia, without acute infarction, where the neurological dysfunction resolves within 24 hours. New-onset renal failure is defined as a 3-fold rise in serum creatinine, creatinine exceeding 4.0 mg/dL with a minimum rise of 0.5 mg/dL, or new need for hemofiltration. A composite outcome measurement has been utilized, defined as patients experiencing any of the following: mortality at 30 days, CVA, TIA, new heart failure/renal failure, or reoperation for bleeding. We have previously described our operative techniques in detail.^7^

### Statistical Analysis

Proportions are described with absolute numbers and percentages, and compared with χ^2^ test, with a continuity correction for 2 × 2 tables, or the Fisher exact test, where cell counts were low. Continuous variables are described with median and interquartile range, and univariate comparisons were performed with the Mann-Whitney *U* test. Multivariable logistic regression was used to adjust the effect value of out-of-hours surgery on the composite outcome for any other significant casemix variables. Model covariates were selected either by identifying those that demonstrated significant differences at a univariate level, or those deemed clinically significant by the research clinicians. The following parameters were offered to the model: age, gender, previous myocardial infarction, respiratory disease, current smoker, renal dysfunction, left ventricular ejection fraction 30% to 50%, and left ventricular ejection fraction <30%. A backward stepwise approach was used for variable selection. *P* value of <.05 is considered statistically significant. Data were analyzed with SAS version 9.4 (SAS Institute Inc, Cary, NC).

## Results

**Figure 1:**
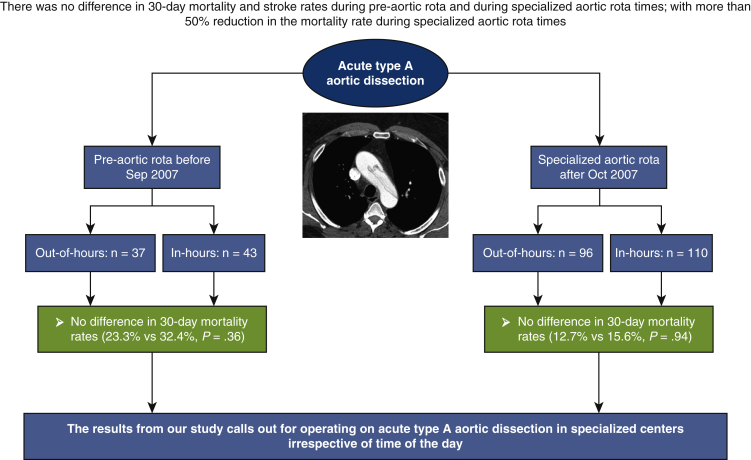
Reported outcomes for patients who underwent repair of acute type A aortic dissection during in hours (8:00 am-7:59 pm) versus out of hours (8:00 pm-7:59 am) during prerota times (October 1998-September 2007) and within the specialized aortic rota times (between October 2007 and December 2019). There was no difference in stroke and 30-day mortality rate between in-hours and out-of-hours surgery.

**Figure 2:**
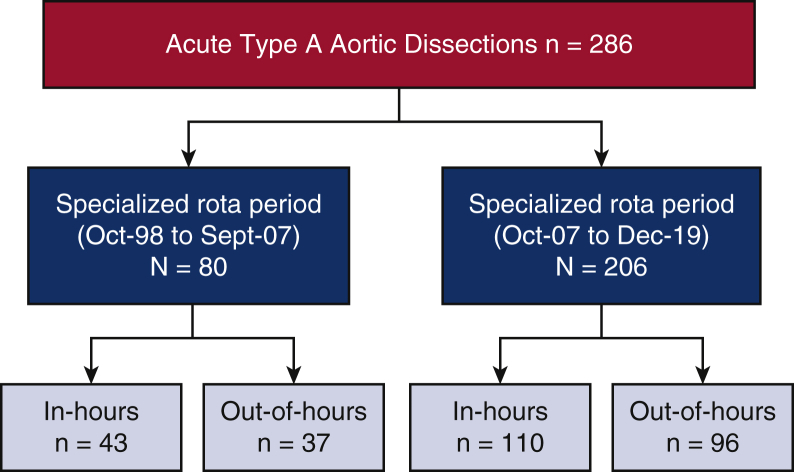
Case distribution between prerota times and specialized aortic rota.

Between October 1998 and December 2019, we identified 286 patients who underwent surgical repair for ATAAD. Eighty of these operations took place during the prerota period; of these 80 cases, 43 operations took place in hours and 37 out of hours. Two hundred six operations took place between SAR periods; 110 operations took place in hours and 96 out of hours (see Figures 1 and 2).

### Patient Demographic Characteristics

During the prerota period, the in-hours and out-of-hours groups were evenly matched in terms of past medical history of diabetes, hypertension, ischemic heart disease, and previous cardiac surgery. There was a significantly longer median duration between presentation and surgery in the in-hours group (585 minutes) compared with the out-of-hours group (185 minutes) (*P* < .001).

During the SAR period, the in-hours and out-of-hours groups had similar rates of diabetes and ischemic heart disease. There was a higher rate of hypertension (63.6% vs 50.0%; *P* = .048) and previous cardiac surgery (7.3% vs 1.0%; *P* = .04) in the in-hours group compared with the out-of-hours group. The SAR period also showed an increased time between presentation and surgery in the in-hours group than the out-of-hours group (592 minutes vs 233 minutes; *P* < .001). Comprehensive patient demographic characteristics are presented in Table 1 and cumulative pre-rota versus SAR patient baseline characteristics are summarized in Table E1.

**Table 1:** Patient demographic characteristics for prerota and specialized aortic rota time periods, stratified by operative time

Characteristic	Pre-rota time period			Specialized aortic rota time period		
	In hours (n = 43)	Out of hours (n = 37)	*P* value	In hours (n = 110)	Out of hours (n = 96)	*P* value
Age at operation (y)	63 (53-69)	62 (53-67)	.66	63 (48-72)	60 (52-69)	.54
Female	9 (20.9)	8 (21.6)	.94	33 (30.0)	40 (41.7)	.08
Body mass index	26.4 (24.1-28.2)	27.5 (24.7-29.4)	.22	27.8 (24.5-32.0)	27.5 (24.0-31.0)	.43
Angina class IV	4 (9.3)	4 (10.8)	>.99	11 (10.0)	4 (4.2)	.11
Previous myocardial infarction	2 (4.7)	2 (5.4)	>.99	9 (8.2)	3 (3.1)	.12
Myocardial infarction within the last 90 d	1 (2.3)	1 (2.7)	>.99	8 (7.3)	2 (2.1)	.11
New York Heart Association functional class ≥ III	7 (16.3)	5 (13.5)	.73	12 (10.9)	10 (10.4)	.91
Current smoker	10 (23.3)	13 (35.1)	.24	20 (18.2)	22 (22.9)	.40
Diabetes	1 (2.3)	0 (0)	>.99	7 (6.4)	4 (4.2)	.48
Respiratory disease∗	10 (23.3)	6 (16.2)	.43	18 (16.4)	11 (11.5)	.31
Hypercholesterolemia	11 (25.6)	5 (13.5)	.18	32 (29.1)	19 (19.8)	.12
Hypertension	30 (69.8)	26 (70.3)	.96	70 (63.6)	48 (50.0)	.048
Previous stroke	4 (9.3)	4 (10.8)	>.99	6 (5.5)	7 (7.3)	.59
Peripheral vascular disease	4 (9.3)	4 (10.8)	>.99	8 (7.3)	5 (5.2)	.54
Renal dysfunction†	3 (7.0)	6 (16.2)	.29	24 (21.8)	17 (17.7)	.46
Left ventricular ejection fraction 30%-50%	6 (14.0)	3 (8.1)	.49	16 (14.6)	11 (11.5)	.51
Left ventricular ejection fraction <30%	2 (4.7)	1 (2.7)	>.99	5 (4.6)	2 (2.1)	.45
Ischemic heart disease	6 (14.0)	1 (2.7)	.12	11 (10.0)	4 (4.2)	.11
Previous cardiac surgery	3 (7.0)	1 (2.7)	.62	8 (7.3)	1 (1.0)	.04
Malperfusion	0 (0)	0 (0)	–	2 (1.2)	8 (8.3)	.05
Time from presentation to surgery (min)	585 (190-1298)	185 (130-301)	<.001	592 (200-1244)	233 (167-339)	<.001

Values are presented as median (range) or n (%).

∗ Respiratory disease was defined as patients having forced expiratory volume in 1 second <75%, asthma, emphysema, chronic obstructive airway disease, or being on respiratory medications.

† Renal dysfunction was defined as patients with a functioning renal transplant and patients with acute or chronic renal failure or insufficiency.

### Operative Details

During the prerota period, there was no difference in the extent of procedures performed or operative times, such as cardiopulmonary bypass time, aortic crossclamp time, or duration of circulatory arrest, between the in-hours and out-of-hours groups.

During the SAR period, there was higher rate of aortic root repairs in the out-of-hours cohort compared with the in-hours cohort (47.9% vs 32.7%; *P* = .03). This difference is likely explained by our adoption of a tear-orientated approach and we do a more extensive surgery such as root replacement or total arch with or without frozen elephant trunk guided by the position of the intimal tear and presence of aneurysmal disease, which is influenced by age and syndromic disease. Operative times were similar, although a slightly shorter median duration of circulatory arrest was seen in the out-of-hours group compared with the in-hours group (46 minutes vs 52 minutes; *P* = .02). Presence of longer operative times in the SAR times is the reflection of surgeon's approach in adopting the practice of reinforcing most of the suture lines with polytetrafluoroethylene or pledgetted sutures that contributed to significant reduction in the mortality and improvement in the reported outcomes during SAR times. Operative details are presented in Table 2.

**Table 2:** Intraoperative variables for prerota and specialized aortic rota time periods, stratified by operative time

Variable	Prerota time period			Specialized aortic rota time period		
	In hours (n = 43)	Out of hours (n = 37)	*P* value	In hours (n = 110)	Out of hours (n = 96)	*P* value
Aortic root	9 (20.9)	9 (24.3)	.72	36 (32.7)	46 (47.9)	.03
Frozen elephant trunk	0 (0)	0 (0)	na	18 (16.4)	8 (8.3)	.08
With or without total arch	16 (37.2)	12 (32.4)	.66	27 (24.6)	22 (22.9)	.78
CPB time (min)	279 (220-355)	318 (261-357)	.17	341 (283-418)	349 (285-415)	.69
AXC time (min)	141 (112-182)	152 (126-204)	.31	199 (148-262)	205 (154-251)	.84
Circulatory arrest time	36 (28-56)	45 (31-67)	.31	52 (42-74)	46 (35-59)	.02

Values are presented as median (range) or n (%). *na*, Not available; *CPB*, cardiopulmonary bypass; *AXC*, aortic crossclamp.

### Postoperative Morbidity, Length of Stay, and 30-day Mortality

During the prerota period, there was no difference in postoperative CVA, TIA, renal failure, or reoperation for bleeding between the in-hours and out-of-hours groups. Total length of hospital and ICU stay was also similar between the 2 groups. Thirty-day mortality was higher in the out-of-hours group than the in-hours group; however, this did not reach statistical significance (32.4% vs 23.3%; *P* = .36).

During the SAR period, rates of postoperative complications, duration of ICU stay, and overall length of hospitalization were similar between for both the in-hours and out-of-hours groups. There was no difference in 30-day mortality between the groups (11.6% vs 11.5%; *P* = .94). Reoperation for bleeding was higher in the in-hours group compared with the out-of-hours group, but this difference was not statistically significant (16.4% vs 9.4%; *P* = .14).

When comparing the prerota period with the SAR period, on average the number of cases per year increased by 83%. A reduction in 30-day mortality was seen over time in both the in-hours (23.3% vs 11.6%) and out-of-hours groups (32.4% vs 11.5%). A reduction in rates of renal failure was also seen during the SAR period, in both the in-hours (23.3% vs 15.5%) and out-of-hours (29.7% vs 17.7%) groups. The median duration of ICU stay was also longer in the SAR period compared with the prerota period, 9 days versus 4 days in the in-hours groups and 7 days versus 4 days in the out-of-hours groups; this is likely due to the higher mortality rate in the prerota times that contributed to shorter ICU stay. The overall postoperative outcomes are presented in Table 3.

**Table 3:** Postoperative outcomes for prerota and specialized aortic rota time periods, stratified by operative time

Outcome	Prerota time period			Specialized aortic rota time period		
	In hours (n = 43)	Out of hours (n = 37)	*P* value	In hours (n = 110)	Out of hours (n = 96)	*P* value
Composite outcome∗	23 (53.5)	20 (54.1)	.96	43 (39.1)	38 (39.6)	.94
30-d mortality	10 (23.3)	12 (32.4)	.36	13 (11.6)	11 (11.5)	.94
CVA	8 (18.6)	5 (13.5)	.54	14 (12.7)	15 (15.6)	.55
TIA	0 (0)	1 (2.7)	.46	1 (0.9)	3 (3.1)	.34
Reoperation for bleeding/tamponade	6 (14.0)	4 (10.8)	.75	18 (16.4)	9 (9.4)	.14
New hemofiltration/renal failure	10 (23.3)	11 (29.7)	.51	17 (15.5)	17 (17.7)	.66
ICU stay (d)	4 (2-7)	4 (1-7)	.67	9 (4-15)	7 (5-21)	.95
Total hospital stay (d)	15 (8-24)	11 (6-18)	.22	16 (10-29)	14 (9-28)	.55

Values are presented as median (range) or n (%). *CVA*, Cerebrovascular accident; *TIA*, transient ischemic attack; *ICU*, intensive care unit.

∗ Composite outcome, defined as patients experiencing any of the following: mortality at 30-days, CVA, TIA, new heart failure/renal failure, or reoperation for bleeding.

It is important to note that the composite end point outcomes did not differ during in-hours versus out-of-hours operating on ATAAD during the prerota and SAR times (Table 3). A further logistics regression analysis showed no different in composite outcomes (odds ratio [OR], 1.20; 95% confidence interval [CI], 0.74-1.97; *P* = .46), and the only dependent variables were female sex (*P* < .001), presence of preexisting respiratory disease (*P* = .02), or poor left ventricular systolic function (*P* = .03) (Table E2).

## Discussion

In this retrospective study, we sought to determine the effect of out-of-hours treatment on outcomes for patients undergoing surgery for ATAAD at our center. Two hundred eighty-six cases were identified, with a fairly even split between the in-hours and out-of-hours groups. The patient demographic characteristics were similar between the in-hours and out-of-hours cohorts in both the prerota and SAR periods. We concluded that there was no major difference in postoperative complications or 30-day mortality rates between our in-hours and out-of-hours cohorts, suggesting that operative quality and standards of care are not influenced by operative timing. Almost a 10% difference in 30-day mortality was found between the in-hours (23.3%) and out-of-hours (32.4%) groups during the prerota period, but this difference was not statistically significant.

**Figure E1:**
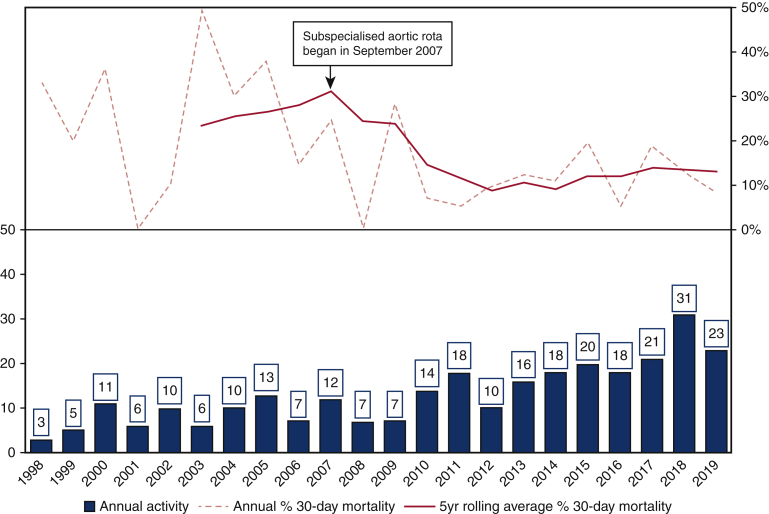
Panel chart showing increase in activity of acute type A aortic dissection repair and reduction in 30-day mortality.

The mean number of cases per year increased by 83% during the SAR period when compared with the prerota period. Since the introduction of a dedicated aortic on-call rota, 30-day mortality reduced in both the in-hours (23.3% vs 11.6%) and out-of-hours (32.4% vs 11.5%) groups, highlighting the importance of high-volume, dedicated aortic surgeons in relation to outcomes and this is contributed to the immediate effect of aortic surgeons performing all the elective and emergency aortic cases (Figure E1).^9^ Total length of hospitalization was similar during both the prerota and SAR periods; however, the median length of ICU stay was longer during the postrota period. Further analysis would be needed to determine whether there is a relationship between length of ICU stay and postoperative mortality, but this is beyond the scope of this article.

A multicenter UK observational study reported higher in-hospital mortality rates in patients undergoing surgery for ATAAD by lower-volume surgeons.^10^ These results were confirmed in a recent systematic review by Mariscalco and colleagues,^3^ which describes a survival benefit in patients treated for acute aortic syndromes by high-volume centers or surgeons (OR, 0.51; 95% CI 0.46-0.56 and OR, 0.41; 95% CI, 0.25-0.66, respectively). Our results were in line with these findings, and we observed a decrease in 30-day mortality rates as center volume increased. Similarly, the introduction of a specialist aortic service at our center was also seen to improve mortality rates both in hours and out of hours. Other centers have demonstrated how the introduction of a multidisciplinary thoracic aortic surgery program and even a multicenter on-call rotational service across 3 hospitals has led to improvements in mortality rates.^11^^,^^12^ Our study compared outcomes of ATAAD during in-hours versus out-of-hours in the presence and absence of SAR, a direction that has not been reported in previous studies that focused on reporting outcomes of in-hours versus out-of-hours only.^11^^,^^12^

There was no difference in major complication or mortality rates between our in-hours and out-of-hours cohorts. There is an established association between weekend admissions and an increased risk of mortality in the literature^13^ and a single-center study by Qiu and colleagues^4^ revealed vast differences in mortality rates between out-of-hours and in-hours surgery for ATAAD (6.42% vs 12.08%; *P* < .05). However, it is likely that the structure of cardiac surgical departments differs both regionally and internationally and so results from single centers may vary. Large national and international database studies have found no such association.^6^^,^^14^ Results from the IRAD database revealed similar rates of major complications and rates of in-hospital and 5-year mortality between in-hours and out-of-hours groups undergoing surgery for ATAAD.^6^ The authors suggested that because their study contained data from several high-volume centers, with highly trained and specialized staff, it is unlikely that resources differed during the out-of-hours period.

At our center, we identified that operative timing for ATAAD did not influence patient outcomes, with no significant differences seen in major complications or 30-day mortality. However, we recognize that at our specialist tertiary center there may be no discrepancy in resources between in-hours and out-of-hours periods. Where higher mortality rates have been noted out of hours, the cause is likely multifactorial and possible explanations include a reduction in staffing levels and experienced staff as well as the availability of diagnostic and therapeutic resources. As seen in our results, other studies have also identified a shorter duration in time from presentation to surgery out of hours.^6^ The severity of illness at admission may therefore vary and patients presenting out of hours are more clinically unstable, requiring more immediate surgical treatment.^15^ Further studies are needed to examine postoperative outcomes following surgery in both in hours and out of hours for ATAAD.^15^, ^16^, ^17^, ^18^ Centers should report on the structure of their aortic service and out-of-hours on-call dissection team as well as independent preoperative predictors of mortality as identified on analysis of the IRAD database (eg, history of aortic valve replacement, migrating chest pain, hypotension, shock or tamponade, cardiac tamponade, and limb ischemia).^15^

### Limitations

This study was conducted as a single-center retrospective analysis on a subset of patients and therefore it is likely that results are influenced by confounders and bias due to the retrospective nature of the study. A further limitation is the lack of recording of patients who were turned down for surgery; this cohort could have been part of this study to assess the importance of service reconfiguration and centralization of aortic service provision.

## Conclusions

Outcomes in repair of ATAAD during in hours and out of hours are similar when the operation takes place in a specialized unit with a dedicated aortic team. This emphasizes the current global trend of service centralization without particular attention to time of day to operate on such critical cohort patients.

### Conflict of Interest Statement

The authors reported no conflicts of interest.

The *Journal* policy requires editors and reviewers to disclose conflicts of interest and to decline handling or reviewing manuscripts for which they may have a conflict of interest. The editors and reviewers of this article have no conflicts of interest.

## Figures and Tables

**Table E1:** Patient demographic characteristics for prerota and specialized aortic rota time periods

Characteristic	Prerota (n = 80)	Postrota (n = 206)	*P* value
Age at operation (y)	63 (53-67)	61 (51-72)	.53
Female	17 (21.3)	73 (35.4)	.02
BMI	27.0 (24.3-29.3)	27.7 (24.2-31.2)	.12
Angina class IV	8 (10.0)	15 (7.3)	.45
Previous myocardial infarction	4 (5.0)	12 (5.8)	>.99
Myocardial infarction within the last 90 d	2 (2.5)	10 (4.9)	.52
NYHA class ≥ III	12 (15.0)	22 (10.7)	.31
Current smoker	23 (28.8)	42 (20.4)	.13
Diabetes	1 (1.3)	11 (5.3)	.19
Respiratory disease∗	16 (20.0)	29 (14.1)	.22
Hypercholesterolemia	16 (20.0)	51 (24.8)	.39
Hypertension	56 (70.0)	118 (57.3)	.048
Previous stroke	8 (10.0)	13 (6.3)	.28
Peripheral vascular disease	8 (10.0)	13 (6.3)	.28
Renal dysfunction†	9 (11.3)	41 (19.9)	.08
Left ventricular ejection fraction 30%-50%	9 (11.3)	27 (13.1)	.67
Left ventricular ejection fraction <30%	3 (3.8)	7 (3.4)	>.99
Ischemic heart disease	7 (8.8)	15 (7.3)	.68
Previous cardiac surgery	4 (5.0)	9 (4.4)	.76

Values are presented as median (range) or n (%). *BMI*, Body mass index; *NYHA*, New York Heart Association.

∗ Respiratory disease was defined as patients having forced expiratory volume in 1 second <75%, asthma, emphysema, chronic obstructive airway disease, or being on respiratory medications.

† Renal dysfunction was defined as patients with a functioning renal transplant and patients with acute or chronic renal failure or insufficiency.

**Table E2:** Logistic regression results for acute type A aortic dissection population, with the composite outcome measure as the dependent variable

Covariate	Odds ratio (95% confidence interval)	*P* value
Out-of-hours surgery	1.20 (0.74-1.97)	.46
Female	0.34 (0.20-0.60)	<.001
Respiratory disease	2.31 (1.17-4.56)	.02
Left ventricular ejection fraction 30%-50%	2.25 (1.07-4.74)	.03

## References

[1] Erbel R., Aboyans V., Boileau C., Bossone E., Di Bartolomeo R., Eggebrecht H. (2014). 2014 ESC guidelines on the diagnosis and treatment of aortic diseases. Eur Heart J.

[2] Hiratzka L.F., Bakris G.L., Beckman J.A., Bersin R.M., Carr V.F., Casey D.E. (2010). 2010 ACCF/AHA/AATS/ACR/ASA/SCA/SCAI/SIR/STS/SVM guidelines for the diagnosis and management of patients with thoracic aortic disease: executive summary. Circulation.

[3] Mariscalco G., Maselli D., Zanobini M., Ahmed A., Bruno V.D., Benedetto U. (2018). Aortic centres should represent the standard of care for acute aortic syndrome. Eur J Prev Cardiol.

[4] Qiu J., Zhang L., Luo X., Gao W., Liu S., Jiang W. (2018). Higher mortality in patients undergoing nighttime surgical procedures for acute type A aortic dissection. Ann Thorac Surg.

[5] Isaac E., Habib A., Hussain A., Crispi V., Chaudhry M., Loubani M. (2019). Does the timing of acute type A aortic dissection surgery impact on immediate and long term patient outcomes? A retrospective single centre study. Clin Surg.

[6] Arnaoutakis G., Bianco V., Estrera A.L., Brinster D.R., Ehrlich M.P., Peterson M.D. (2020). Time of day does not influence outcomes in acute type A aortic dissection: results from the IRAD. J Card Surg.

[7] Bashir M., Shaw M., Field M., Kuduvalli M., Harrington D., Fok M. (2016). Repair of type A dissection-benefits of dissection rota. Ann Cardiothorac Surg.

[8] Society of Thoracic Surgeons STS Adult Cardiac Surgery Database data specifications. https://www.sts.org/sites/default/files/ACSD_DataSpecifications_V4_20_2.pdf.

[9] Chikwe J., Cavallaro P., Itagaki S., Seigerman M., Diluozzo G., Adams D.H. (2013). National outcomes in acute aortic dissection: influence of surgeon and institutional volume on operative mortality. Ann Thorac Surg.

[10] Bashir M., Harky A., Fok M., Shaw M., Hickey G.L., Grant S.W. (2017). Acute type A aortic dissection in the United Kingdom: surgeon volume-outcome relation. J Thorac Cardiovasc Surg.

[11] Andersen N.D., Ganapathi A.M., Hanna J.M., Williams J.B., Gaca J.G., Hughes G.C. (2014). Outcomes of acute type A dissection repair before and after implementation of a multidisciplinary thoracic aortic surgery program. J Am Coll Cardiol.

[12] Vaja R., Talukder S., Norkunas M., Hoffman R., Nienaber C., Pepper J. (2019). Impact of a streamlined rotational system for the management of acute aortic syndrome: sharing is caring. Eur J Cardiothorac Surg.

[13] Freemantle N., Ray D., McNulty D., Rosser D., Bennett S., Keogh B.E. (2015). Increased mortality associated with weekend hospital admission: a case for expanded seven day services?. BMJ.

[14] Ahlsson A., Wickbom A., Geirsson A., Franco-Cereceda A., Ahmad K., Gunn J. (2019). Is there a weekend effect in surgery for type A dissection?: results from the Nordic Consortium for acute type A aortic dissection database. Ann Thorac Surg.

[15] Trimarchi S., Nienaber C.A., Rampoldi V., Myrmel T., Suzuki T., Mehta R.H. (2005). Contemporary results of surgery in acute type a aortic dissection: the International Registry of Acute Aortic Dissection experience. J Thorac Cardiovasc Surg.

[16] Toh S., Yew D.C.M., Chong T.L., Harky A. (2020). Acute type A aortic dissection in-hours versus out-of-hours: a systematic review and meta-analysis. J Card Surg.

[17] Harky A., Singh V.P., Khan D., Sajid M.M., Kermali M., Othman A. (2020). Factors affecting outcomes in acute type A aortic dissection: a systematic review. Heart Lung Circ.

[18] Saw L.J., Lim-Cooke M.S., Woodward B., Othman A., Harky A. (2020). The surgical management of acute type A aortic dissection: current options and future trends. J Card Surg.

